# Hypotensive Patient Presenting With Abnormal Pre-hospital Ischemic Electrocardiogram: A Case of Pulmonary Embolism Diagnosed by Point-of-Care Ultrasound (POCUS)

**DOI:** 10.7759/cureus.87909

**Published:** 2025-07-14

**Authors:** Caleb Ellis, Jared Wenn, Jordan Brunswick, Justin Lake, Hillary McKinley

**Affiliations:** 1 Emergency Medicine, Wright State University, Dayton, USA; 2 Emergency Medicine, Miami Valley Emergency Specialists, Dayton, USA

**Keywords:** critical care, emergency medicine, pocus, pulmonary emboli, stemi

## Abstract

Electrocardiograms (ECGs) are widely utilized to identify a variety of emergent and life-threatening conditions and are routinely used in the pre-hospital setting. Early and accurate identification of cardiac conditions such as ischemia or arrhythmia can facilitate accurate and prompt medical management by the pre-hospital team and the emergency department providers. This case describes a 65-year-old male with a pre-hospital presentation of chest pressure and a syncopal event. A pre-hospital ECG was concerning for ST-segment elevation myocardial infarction (STEMI), but the patient was ultimately found to have an alternative diagnosis of pulmonary embolism (PE) with an intraventricular thrombus identified on cardiac point-of-care ultrasound (POCUS). ECGs and POCUS should be jointly utilized in the patient assessment to consider a broad differential diagnosis as there are alternative pathologies that can mimic STEMI-like ECG changes. Treatment of the underlying pulmonary emboli with thrombolytic therapy led to stabilization of this patient and ultimately led to the patient being discharged home from the hospital. At a four-month follow-up appointment, the patient remained on oral anticoagulation, and a routine echocardiogram demonstrated normal ventricular size and function.

## Introduction

Chest pain is one of the most common chief complaints of patients presenting to the Emergency Department (ED), with over 7 million annual visits in the United States [1]. ECGs can identify cardiac and non-cardiac related conditions [2]. Early and accurate identification of cardiac conditions such as ischemia, arrhythmia, heart block, right ventricular heart strain, pericarditis, or cardiac tamponade can facilitate accurate and prompt medical management by the pre-hospital team, the emergency department providers, and specialty consultants. However, ECGs in isolation are rarely diagnostic and need to be appropriately paired with the clinical presentation as well as additional laboratory and imaging results. Lab results take time to process after collection, and several established guidelines are built upon trending troponin values to rule in or rule out pathology such as acute coronary syndrome [3,4]. Point-of-care ultrasound (POCUS) is instrumental whereby the provider is simultaneously performing and interpreting the sonographic images to gain real-time information to optimize patient care. This case describes a patient with chest pressure and a syncopal event that had a pre-hospital ECG initially concerning for ST-segment elevation myocardial infarction (STEMI), however, the patient was promptly found to have an alternative diagnosis of pulmonary embolism (PE) with an intraventricular thrombus as identified on cardiac POCUS. After discussion with the interventional cardiologist, alteplase was administered for the treatment of the underlying PE, which led to hemodynamic stabilization. In this case, POCUS rapidly identified the underlying etiology responsible for the ECG changes and the patient was given thrombolytic therapy, avoiding a delay in care as cardiac catheterization was not indicated despite ECG changes [5]. ECGs and POCUS should be jointly utilized in the patient assessment to consider a broad differential diagnosis to help facilitate early identification and treatment of emergent and life-threatening conditions [6-8].

## Case presentation

**Figure 1:**
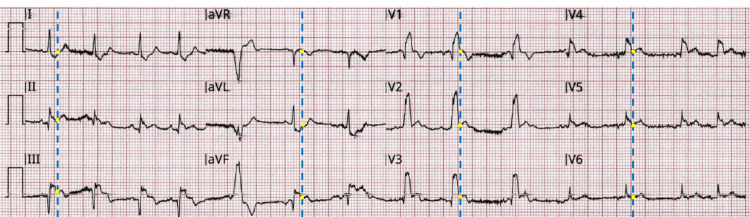
Pre-hospital ECG obtained by medics. Blue dashed line overlaid to demonstrate consistency of J point (yellow dot) assessment.

A 65-year-old male with no significant past medical history presented to the emergency department via ambulance with concern for chest pressure and a syncopal episode. Further history elicited that the patient was employed in a sedentary job, had a cough for the last 4 weeks with associated shortness of breath, and a prolonged period of inactivity compared to his baseline sedentary lifestyle. The medics found the patient cool and diaphoretic with an initial blood pressure (BP) 40/22 mmHg and a heart rate (HR) of 105 beats per minute. Medics were concerned that the initial pre-hospital ECG demonstrated an inferior STEMI pattern (Figure 1) and a STEMI alert was activated. Medics provided 324 mg of aspirin but were unable to obtain intravenous access prior to arrival, so no sublingual nitroglycerin was administered. On arrival to the emergency department, the patient had critically abnormal vital signs (HR 107 bpm, BP 75/41 mmHg, oxygen saturation 81% on room air, which improved to 100% oxygen saturation on 15 liters via non-rebreather mask, rectal temperature 95.1° F). The initial presenting BP could have been elevated compared to the pre-hospital vital signs from moving the patient over to the hospital gurney, since the subsequent blood pressures were lower, decreasing to 40/22 mmHg on the subsequent assessment three minutes later. The pre-hospital ECG was thought to represent a new right bundle branch block (RBBB) pattern by interventional cardiology, but there was no prior ECG available for comparison.

Figure 1 demonstrates an abnormal ECG with evidence of a right bundle branch block. The QRS complex is widened at 154 milliseconds. When assessing the J point (yellow dot) in each of the above leads, there is J point elevation in Lead III and V3 (dashed line in Figure 1) when compared against the T-P baseline. There is also J point depression in aVL. However, this does not meet the definition of STEMI as there is no J point elevation in two anatomically contiguous leads, but J point elevations can be noted in RBBB. Right bundle branch patterns can result from myocardial ischemia, pulmonary emboli, volume overload, pulmonary hypertension, congenital heart defects, or myocarditis among others [2].

**Figure 2:**
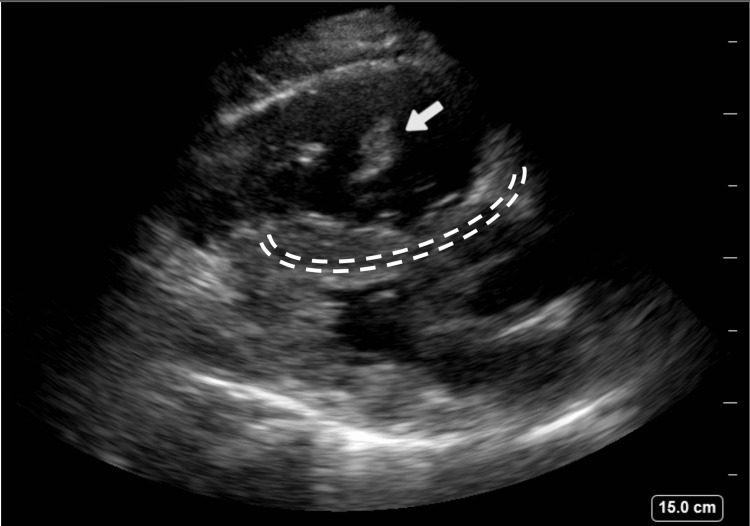
Sonosite XPorte with phased array probe in a parasternal long axis view demonstrating a mobile hyperechoic structure (white arrow) in the right ventricle. Interventricular septal bowing from right to left is visible (white dashed lines).

**Figure 3:**
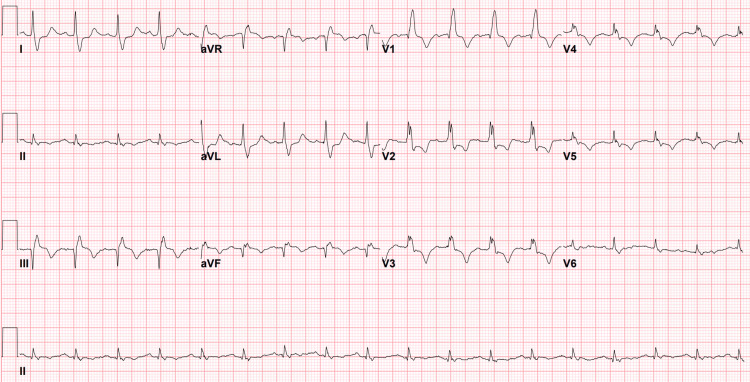
ECG after alteplase administration demonstrating S-waves in lead I, Q-waves and inverted T-waves in lead III (S1Q3T3) as well as a continued right bundle branch block pattern and deeply inverted T waves in the precordial leads.

**Figure 4:**
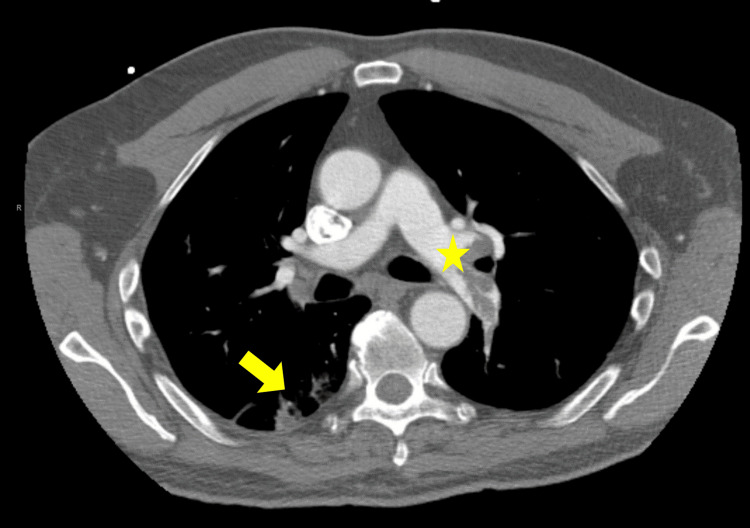
Transverse CT scan with contrast showing filling defect (star) in the left pulmonary arteries with right posterior pulmonary infarct (arrow).

The patient was in uncompensated cardiopulmonary shock as demonstrated by diaphoresis, tachycardia, hypotension, and hypoxia. Intravenous access was obtained and fluid resuscitation with one liter of normal saline was initiated. The pre-hospital ECG prompted the use of cardiac POCUS. The emergency medicine resident was able to rapidly obtain parasternal short and long axis cardiac views with the phased array probe showing a mobile hyperechoic area in the dilated right ventricle, raising suspicion of pulmonary embolism (Figure 2 and Video 1). No further cardiac views or ultrasound images were obtained. At this time, the patient had completed the liter of normal saline and was initiated on an infusion of norepinephrine at 0.2 mcg/kg/min for hemodynamic support due to persistent hypotension with a mean arterial pressure less than 65 mmHg. After discussion with the interventional cardiologist and with POCUS demonstrating concern for a massive pulmonary embolism with mobile thrombus, the decision was made to administer thrombolytics after ruling out absolute contraindications [9]. Alteplase 100 mg intravenous over 2 hours was administered. Repeat ECG after thrombolytic therapy demonstrated an S1Q3T3 pattern (Figure 3). The patient’s hemodynamics began to stabilize about 20 minutes after initiation of alteplase (HR 122, BP 100/50 with mean arterial pressure 72), and the oxygen was weaned down to 5 L nasal cannula a little less than 2 hours after initiation of alteplase. Initial labs were significant for a mixed acidosis on venous blood gas (pH 7.13, pCO2 51.7, HCO3 17), BNP 371 pg/mL, lactate 9.6 mmol/L, Troponin T 26 ng/L with a 1-hour Troponin T 2,058 ng/L (Delta 7,815). The patient was admitted to the intensive care unit (ICU) and started on a continuous heparin infusion. He underwent ultrasound venous Doppler of bilateral lower extremities and was found to have a deep vein thrombus (DVT) of the left femoral vein. A limited transthoracic echocardiogram with Doppler and color demonstrated an estimated ejection fraction of 55-60% with grade I diastolic dysfunction, moderate right ventricular dilation and mild dilation of bilateral atrium. The subsequent day, the patient underwent computer tomography (CT) scanning with contrasted examination of the pulmonary arteries that demonstrated bilateral pulmonary emboli of both main pulmonary arteries with evidence of widening of the right ventricle, suggestive of right heart strain, as well as bilateral pulmonary infarcts (Figure 4) as interpreted by the radiologist. In the setting of clinical improvement with patency noted through all pulmonary arteries despite residual clot, thrombectomy was not recommended by the consulting cardiologist. Hematology was consulted and recommended transition from heparin infusion to oral apixaban. He was discharged home on hospital day seven to continue oral apixaban.

**Video 1:** Sonosite XPorte with phased array probe in a parasternal long axis view demonstrating a mobile hyperechoic structure in the right ventricle (RV), which is bowing towards the left ventricle (LV). The left ventricular outflow tract (LVOT) is also visible.

## Discussion

In the setting of an unstable patient with chest pain and a syncopal episode combined with an ECG concerning for ST-segment elevation in several leads, acute coronary syndrome (ACS) must be considered in the differential diagnosis. However, closer inspection reveals the ECG is not consistent with STEMI criteria. Although the pre-hospital emergency medicine services personnel appropriately identified an abnormal ECG and requested a cardiac alert with concern for acute myocardial infarction, an alternative diagnosis was quickly found in the ED. It is important to note that laboratory testing and advanced imaging modalities are inherently not available in the pre-hospital setting.

This case highlights the need to refrain from diagnostic anchoring and rather to rely on the clinical presentation in combination with laboratory testing and advanced POCUS and CT imaging, as the STEMI-mimic was ultimately attributed to a massive PE. ECG findings of PE may include patterns such as the aforementioned S1Q3T3, any right bundle branch block, right axis deviation, tachycardia, or non-specific T wave inversions in the septal precordial leads. While a new RBBB is not specific for pulmonary emboli, if a RBBB is present in the setting of pulmonary emboli, it may be used as a sign of main pulmonary artery obstruction or saddle emboli [5]. However, none of those possible ECG findings are sensitive or specific to be clinically reliable [10]. In addition, if this was a true ACS event, the providers may have been able to see regional wall motion abnormalities (RWMAs), which are known to precede ECG changes [6]. POCUS assessment of RWMAs is most commonly performed as a POCUS assessment in the parasternal short axis or the apical four-chamber views to assess hypokinetic left ventricular wall motion presumably due to occlusion of blood flow. In this case, RWMA would have been less helpful as a bundle branch block as well as valvular pathology and cardiomyopathy, among other causes, can also lead to this appearance of hypokineses [11]. Without having a prior ECG or prior echocardiogram to compare, RWMA in isolation would not have been diagnostic in this particular case. There are several signs of right heart strain associated with a diagnosis of PE, such as the right ventricular bowing that was previously described in Figure 2, as well as right ventricular dilation relative to the left ventricle [12]. However, seeing mobile thrombus within the right ventricle was pathognomonic for a large embolism; therefore, the patient was quickly treated with thrombolytics. There are several pre-hospital STEMI mimics that have been previously documented, and POCUS evaluation in combination with clinical and laboratory testing has been shown to reliably and rapidly assess for alternative diagnoses [7, 8, 13].

## Conclusions

This case demonstrates the diagnostic uncertainty with abnormal ECGs and pre-hospital diagnostic anchoring. The pre-hospital personnel requested an acute myocardial infarction protocol based on the abnormal ECG; however, the ECG is abnormal without meeting true STEMI criteria. Additional information was gathered by utilizing laboratory testing along with POCUS and CT imaging to arrive at the correct diagnosis of a PE. Treating the PE led to hemodynamic stabilization of the patient as evidenced by improvement in mean arterial pressures and decreased supplemental nasal cannula oxygen requirements within 2 hours of receiving thrombolytics. Emergency providers should be aware of cardiac POCUS signs suggestive of PE given that it is an acute life-threatening presentation for thousands of patients every year, and be simultaneously aware of the diagnostic challenges in abnormal ECGs. The prompt identification and treatment of the correct pathology led to a successful medical course for this patient as he has remained without any long-term cardiac pathology noted during his four-month follow-up.
